# Abstracts of the International Conference and Expo on Microbiology. Barcelona, Spain November 18–19, 2019. Hotel Novotel Barcelona Sant Joan Despi

**DOI:** 10.3390/microorganisms8020267

**Published:** 2020-02-18

**Authors:** Irene Frank

**Affiliations:** Microbiology Conference 2020, Coalesced Research Group, 08970 Barcelona, Spain; microbiology@conferencesinsight.com

## Abstract

International Conference and Expo on Microbiology (Microbiology 2019) organized by Coalesce Research Group was held on November 18–19, 2019 at Hotel Novotel Barcelona Sant Joan Despi, Barcelona, Spain. The conference highlighted the theme, “Your Microbe, Your Research”. Benevolent response and active participations were received from the Scientists, Doctors, Researchers, Students, and Leaders from the fields of Microbiology Research, who made this event inspiringly successful.

## Keynote Presentations

### Closing the Gap in Sepsis Diagnostics 

AlavieTinoQvella Corporation, Canada; TinoA@qvella.comSepsis, its severity, and how to manage affected patients is confounding as symptoms are common to many other conditions. The problem is further exacerbated when faced with treatment options in the absence of timely diagnostic information. The presentation will highlight Qvella’s take on the opportunity, challenges, and solutions for addressing this difficult and multivariate problem by delivering timely diagnostic results.

### μAqua: Microarrays to Detect Cyanobacteria and Their Toxins

MedlinL. K.Marine Biological Association of the United Kingdom, United Kingdom; lkm@mba.ac.ukCyanobacteria are oxygenic phototrophic prokaryotes, some of which produce a variety of toxins and pose a serious health threat to drinking water worldwide. The frequency, intensity and distribution of Cyanobacterial blooms in freshwaters have been growing worldwide, with the major causes generally correlated with water eutrophication and climate changes. About 40 species produce potent toxins, potentially causing the so-called Harmful Algal Blooms to impact heavily on environmental and human health. The risk of human exposure comes from contaminated recreational surface waters and from consuming untreated drinking water or contaminated food. To make novel tools for the early and sensitive detection of cyanobacteria and their toxins. A phylochip (microarray), which identifies the presence of freshwater Cyanobacterial targets using rRNA barcodes, and a RT-PCR microarray to amplify the mRNA captured by the barcodes for genes involved in cyanobacterial toxin synthesis were developed. For the phylochip, the presence of Cyanobacteria was detected through the use of rRNA barcodes used in a fluorescent microarray detection platform. For the toxin array, we performed a reverse transcriptase extension of the mRNA probes for various toxin pathways immobilised on the array incorporating fluorescent nucleotides in the reaction to amplify the signal from the messenger RNA that is expressed in low quantities in the cells. The μAQUA phylochip was field tested in six countries with good validation achieved with either cell counts or flow cytometric measurements. The toxin array was tested in two countries, with good validation by chemical means to detect which populations of Cyanobacteria were toxic. When compared to the toxin array, target organisms that could produce the highlighted toxins were present on the phylochip on the same sampling day. The species and the toxin array when used together provide a secure early warning system for cyanobacterial toxin blooms as defined by health and fishery administrators. Preliminary evidence suggests that the toxin array detect expression of toxin genes long before they can be detected by chemical means.

## Plenary Presentations

### Human Milk Oligosaccharides: Antibiofilm Activity against Pathogens Isolated from Cystic Fibrosis Patients

OledzkaGabriela*JarzynkaSylwiaStromKamilaMinkiewiczAnnaBarbarskaOlgaWesolowskaAleksandraMakarewiczOliwiaMedical University of Warsaw, Poland*Correspondence: gabriela.oledzka@wum.edu.plHuman milk oligosaccharides (HMOs) are the third most abundant component of breast milk, after fat and lactose. HMOS act as probiotics shaping the gut microbiome of fed infants by promoting the growth of beneficial bacteria while inhibiting adhesion of pathogenic species. Recent studies have shown that HMOs isolated from human milk samples demonstrated antimicrobial and antibiofilm activity against different strains. Cystic fibrosis (CF) is one of the major respiratory diseases, and the clinical management and definitive treatment of cystic fibrosis (CF) biofilm-mediated chronic bacterial lung infection remains a challenge. In this study, we examine human milk oligosaccharides (HMOs) antibiofilm activity against pathogens isolated from cystic fibrosis patients. In our preliminary works, we investigated the antibiofilm activity of the saccharide fraction obtained from pooled human milk of nine donors against various pathogenic bacteria that were recently categorised as ‘critical priority’ (*Acinetobacter baumannii*, *Pseudomonas aeruginosa*, *Enterobacteriaceae*) and ‘high priority’ (*Staphylococcus aureus*) by the World Health Organization (WHO) but also against *Burkholderia cenocepacia*, an intrinsically multi-resistant pathogen associated with high mortality in Cystic Fibrosis (CF) patients. We tested the ability of HMOs to inhibit biofilm formation and to eradicate matured biofilms. The pooled HMOs showed a biofilm eradicating effect on most tested pathogens. The results obtained by confocal laser scanning microscopy after life/dead staining of untreated (control) and HMOS treated biofilms were shown for selected species with focus on lung infections. The HMOs effectively killed the bacteria at high concentration (20 mg/mL, corresponds to the concentration in human milk), but visible reduction of viable bacteria and biofilm mass was observed already at lower concentrations that varied between the species. The biofilm mass was also reduced in almost all pathogenic biofilms. The data presented in this paper supporting the importance and potential inhibitory effect of HMOs in biofilm formation. HMOs could potentially be used as novel therapeutics to treat or prevent infectious disease in patients with CF.

### New Horizons in Regulation of New Technologies for Veterinary Vaccines

HernandezJavier Martinez de VelascoSpanish Medicinal Products Agency, Spain; jmartinezv@aemps.esConventional vaccinology has been used with successful results against many pathogens over the years but there are many pathogens for which conventional vaccinology has failed. There could be many reasons for this lack of efficacy for “traditional” vaccines and therefore is increasing the need for new technologies for developing new mechanisms of action for the vaccines, new animal models, new delivery mechanisms, and many strategies to change the way vaccines act today. The new changes in technology also need changes in regulation to include the differences that can be seen compared to the conventional vaccines. In words of Guido Rasi (EMA Executive Director) some questions arise with these needs: How ready are we to engage with these emerging science and technological innovations? Do we have the necessary skills and competencies or indeed access to the specific expertise required? Are we generating new guidance or providing sufficient levels of advice to facilitate the utilization and translation of these innovations? For these reasons a consultation for all stakeholders has been launched to enable the EMA to keep on top of developments, identify the gaps between science and healthcare systems and bring together the various stakeholders needed to bridge those gaps. This introduction for the expectations of a future regulation should be seen with the new implemented changes in regulation “New Regulation 2019/6—Veterinary Medicinal Products” (applicable in 2022) including the new technologies and the future development of this regulation. To sum up, some examples of already authorized products that fall into the Advanced Therapies Medicinal Products definition are described. To foster scientific excellence in the regulation of veterinary medicines for the benefit of animal and public health while facilitating and promoting innovation and access to novel medicinal products. The Agency’s final goal in the veterinary, as in the human, sphere is to develop the existing interaction between the EU regulatory network and academia further, in order to be kept informed of relevant scientific innovations and research and anticipate solutions to regulatory needs and challenges. This is the key to delivering the other strategic goals and recommendations laid out in this document. It is envisaged that this aim will be achieved by establishing a novel regulatory science and innovation platform in partnership with academic research centers. The ultimate aim is to ensure that regulatory science remains at the cutting edge so that EMA can deliver its fundamental mission of protecting human and animal health and facilitating the availability of medicines to animals.

### Understanding and Harnessing the Power of Beneficial Bacteria for Sustainable Agriculture and Environment

YuanZe-ChunUniversity of Western Ontario, Canada; zyuan27@uwo.caModern agriculture relies on a high level of chemical input. These include fossil fuels to drive machinery, as well as chemical fertilizers, pesticides, and herbicides—all of which affect both soil quality/biodiversity and ecosystem health. Plant/soil microbes play important roles in mediating the earth’s biogeochemical cycles, enhancing soil fertility, promoting crop productivity and stress tolerance to pathogens and insects. Thus, it has great potential in understanding and harnessing the power of beneficial bacteria for sustainable agriculture and environment. We identified and applied plant/soil bacteria for bioremediation/biodegradation and plant growth promotion. Through screening bacteria growth on minimal medium and in planta experiments, we isolated bacteria that are able to grow with lignin or diesel as the sole carbon source; produce plant growth hormones or antimicrobial compounds and grow with glyphosate (herbicide) as the sole carbon and phosphate source. Currently we are intensively working on *Pseudomonas azotoformans* P45A (GenBank: CP041236) capable of degrading herbicide (glyphosate) and promoting plant growth. In addition, we are integrating environmental microbiology with environmental engineering (electrokinetics) to enhance the bioremediation of diesel and phenanthrene (Hassan et al. *J. of Environmental Science and Health*. 2019; *Chemosphere* 2019; *Bioremediation J*. 2017; *J. of Environmental Chemical Engineering*. 2015; *Genome Announcements* 2015). In total, we isolated and identified over 1200 bacterial strains capable of promoting plant growth (Grady et al. *BMC Microbiology* 2019), degrading lignin (Weselowski et al. *BMC Microbiology* 2019; Eastman et al *Frontiers in Microbiology* 2015), degrading herbicide (glyphosate) or diesel. So far, we completed the genome sequencing for 11 bacterial isolates, followed by intensive genetics and genomics characterization (Eastman et al. *BMC Genomics* 2014). Our researches established that plant/soil bacteria can be utilized as environmentally friendly solutions to address the major challenges of modern agriculture and environmental ussies.

### Investigation of a Physicochemical Association between Various Saccharide and Nanoparticles during Freeze-Drying

KamiyaSeitaro*KabashimaTsutomuNakashimaKenichiroNagasaki International University, Japan*Correspondence: kamiya@niu.ac.jpThere are few reports on the preservation of nanoparticles. Nanoparticle suspensions are thermodynamically unstable and subject to aggregation. Maintaining a constant state in nanoparticles is an important major issue. Freeze-drying on addition of saccharides is a useful method for preventing aggregation. In the present study, trisaccharides, tetrasaccharides, and pentasaccharides was employed as an additive. In addition, we hypothesize the interactive mechanism between stachyose and the nanoparticles during freeze-drying for the first time. The mean particle size of the rehydrated freeze-dried sacchride-containing nanoparticles was similar to the initial particle size before freeze-drying, indicating that the particle size had been maintained. The mean particle size of the rehydrated normal-dried sacchride-containing nanoparticles indicate varied particle sizes. The powder X-ray diffraction of the freeze-dried nanoparticles-containing sacchride revealed a halo pattern. The powder X-ray diffraction of the normally dried nanoparticles-containing stachyose produced several patterns. These results suggest an interaction between the nanoparticles and stachyose, and that this relationship depends on whether the mixture is freeze-dried or dried normally. We studied the association between the nanoparticles aggregation and the crystal form of saccharides and their mechanisms by using the obtained results of the data of particle size, powder X-ray pattern, and DSC curves. The particle size of the nanoparticles was maintained when it was freeze-dried, while particle aggregation occurred when normal dried samples were used. In addition, crystallinity crystalline saccharide was not observed in the in the freeze-dried group but was in the normal dried group.

### Growth of an Anaerobic Sulfate-Reducing Bacterium Sustained by Oxygen Respiratory Energy Conservation after O_2_-Driven Experimental Evolution

DollaAlain*SchoefflerMarineMediterranean Institute of Oceanology, Aix-Marseille University, France*Correspondence: alain.dolla@mio.osupytheas.frDesulfovibrio species are representatives of microorganisms at the boundary between anaerobic and aerobic lifestyles, since they contain the enzymatic systems required for both sulfate and oxygen reduction. However, the latter has been shown to be solely a protective mechanism. By implementing the oxygen-driven experimental evolution of Desulfovibrio vulgaris Hildenborough, we have obtained strains that have evolved to grow with energy derived from oxidative phosphorylation linked to oxygen reduction. We show that a few mutations are sufficient for the emergence of this phenotype and reveal two routes of evolution primarily involving either inactivation or overexpression of the gene encoding heterodisulfide reductase. We propose that the oxygen respiration for energy conservation that sustains the growth of the O_2_-evolved strains is associated with a rearrangement of metabolite fluxes, especially NAD+/NADH, leading to an optimized O_2_ reduction. These evolved strains are the first sulfate-reducing bacteria that exhibit a demonstrated oxygen respiratory process that enables growth.

## Session Speakers

### A High Mutation Rate Underpins the Rapid Acquisition of Multidrug Resistance among Lineage 4 Haarlem Genotype Epidemic Strains, Northern Tunisia

MardassiHelmi*DekhilNairaMeftahiNedraMhenniBesmaFrajSaloua BenUniversity of Tunis El Manar, Tunisia*Correspondence: helmi.merdassi@pasteur.tnUnderstanding how multidrug-resistant tuberculosis (MDR-TB) emerge and spread, particularly in an HIV-negative context, could help deciphering the contributing factors inherent to the bacillus. Here we explored thoroughly the population structure of the *Mycobacterium tuberculosis* lineage 4 (L4)-Haarlem genotype in Northern Tunisia, where it caused from the very outset a major MDR-TB outbreak among HIV-negative young individuals. We ensured an 11-year full coverage of the Haarlem genotype in the epidemic region by analyzing all *M. tuberculosis* isolates displaying the Haarlem spoligotype signature (N = 253). We performed 24-loci MIRU-VNTR (multiple interspersed repetitive unit-variable-number tandem repeat) typing and whole-genome sequenced 94 representative isolates. Various population genetics and evolutionary analyses were then carried out. We disclosed the propensity of the Haarlem genotype to undergo an epidemic spread, irrespective of the drug susceptibility pattern (83% overall recent transmission rate). The overall MDR-TB epidemic was linked to a single drug-susceptible progenitor clone, whose existence was dated back 124 years. Strikingly, an overall high mutation rate close to that of the globally spread L2-Beijing sublineage was estimated, a finding in line with the almost concurrent acquisition of multi-drug resistance and compensatory mutations. Such a high mutation rate also explains the elevated number of nucleotide differences between the genomes of isolates from patients with established epidemiologic direct links. Our data uncovered the relative high mutation rate of the Haarlem genotype evolving in Northern Tunisia, an inherent hallmark that most likely contributed to the emergence of successfully transmitted MDR clones through rapid acquisition of compensatory mutations. If not contained, this rapidly evolving Haarlem clone, which at present remains geographically confined, could represent a threat to the Euro Mediterranean space.

### Defining the Possible Natural Habitat of Acinetobacter baumannii Outside Hospitals

HrenovicJasna^1^*DekicSvjetlana^1^DurnGoran^1^BarisicIvana Goic^2^GanjtoMarin^3^1University of Zagreb, Croatia2University Hospital Centre Split and University of Split School of Medicine3Split Wastewater – Management and Operation Ltd, Croatia*Correspondence: jasna.hrenovic@biol.pmf.hrDue to the development of resistance to last resort antibiotics, bacterium *Acinetobacter baumannii* is nowadays a leading cause of nosocomial outbreaks. Clinically relevant *A. baumannii* were reported outside hospital settings, including natural waters and soils influenced by human waste. Viable* A. baumannii* in nature represent a public-health risk for humans and animals that are exposed to it. The aim of this study was to investigate the survival of *A. baumannii* in natural substrates to define its possible natural habitat outside hospitals. Clinical and environmental but clinically relevant isolates of *A. baumannii*, resistant to carbapenems, fluoroquinolones, and even colistin, were inoculated into different types of waters and soils. The pH and nutrient availability was determined in media employed. Numbers of *A. baumannii* were monitored using CHROMagar Acinetobacter plates with carbapenem-selective supplement CR102 after incubation at 42 °C/48 h. In the treated urban wastewater and river water *A. baumannii* multiplied and survived for 50 days, while no multiplication occurred in the seawater. In the slight alkaline red palaeosol and technosol* A. baumannii* multiplied and survived up to 5 months, but did not multiply in weakly acid Terra Rossa. The multiplication dependent on the nutrient availability was confirmed in the nutrient broth, but was absent in the spring water. Natural waters and soils of near neutral pH rich in nutrients support the multiplication of *A. baumannii*, and are a possible natural habitat of this emergent pathogen outside hospitals. 

### Virulence genes rib and bca in Serotypes of Group B Streptococcus (GBS) Isolated from Symptomatic Pregnant Women in a Tertiary Care Hospital in East Coast Malaysia (61587)

MahmudMohammed Imad A. MIA Mustafa*BahezAyesha AHamzahHairul Aini HA btWahidHanan Hamimi HH btInternational Islamic University Malaysia, Malaysia*Correspondence: imad@iium.edu.myGroup B streptococcus (GBS) is a leading cause of maternally acquired invasive infections in neonates. Maternal immunization with GBS vaccine is of utmost demand for prevention of these infections. Required knowledge concerning vaccine candidates includes serotype-specific polysaccharides and GBS virulence proteins. This study aimed to undertake capsular serotyping and virulence factor genes identification for the local GBS isolates as a pilot study to identify vaccine candidates. Standard microbiological methods, according to the Center for Disease Control and Prevention (CDC) recommendations, were used to identify GBS serotypes. A total of 62 GBS isolates from high vaginal swabs of symptomatic pregnant women were collected from the 1^st^ of March 2018 to 30^th^ of July 2018. The isolates identity was reconfirmed by molecular methods. Latex agglutination test was performed to determine the GBS serotypes according to the specificity of the capsular polysaccharide. Of the 62 examined GBS isolates, 48 were serologically typeable, representing 77.4%, and 14 were serologically non-typeable representing 22.6% of the samples. Serotype Ia and Ib (16.1% each) was the most common capsular types, followed by II, V, and VII (9.7% each), III (8.1%), VI (6.5%), and VIII (1.6 %). Among all 10 GBS serotypes, serotypes IV and IX were not detected in the present study. Real-time PCR revealed that 42 (67.7%) isolates harbored the *rib* gene while 61 (98.4%) isolates harbored the *bca *gene. Our findings showed that the five widely known prevalent serotypes in other regions in the world which are considered as candidates for pentavalent CPS-conjugate vaccine do not match the CPS distribution in symptomatic pregnant women in Kuantan. On the other hand, the frequency of virulence genes* rib* and *bca* is higher in our isolates, which tentatively makes the proteinaceous vaccine, N-terminal domains of Rib and AlpC a more suitable choice for GBS prevention in this geographical area. However, further wider study recruiting a larger number of isolates from various Malaysian states is required to confirm this conclusion. 

### Mimivirus on a Bad Hair Day 

SirkisYael Fridmann*MinskyAviWeizmann Institue of Science, Israel*Correspondence: yael.fridmann-sirkis@weizmann.ac.il*Acanthamoeba polyphaga mimivirus* (APMV) is the first giant virus that was discovered in the past two decades. Mimivirus has various uncommon characteristics to other viruses such as a stargate shape in one of its vertices through which its dsDNA is released into the cytoplasm upon infection. It also has an external thick fibril layer that was shown to be important for adhesion to its host. This finding was based on a mimivirus strain that suffered a drastic reduction in the number of its fibrils after a third of its genes lost activity during continuous passaging in germ-free amoebae. To examine the role of the fibrils surrounding the mimivirus, enrichment of fibril-deficient virus population by subculturing consecutive generations under regular conditions and continually passing them through 0.45 μm filters was undertaken. Genomic analysis of the filtered viruses revealed three mutations that affected only three genes. One of the mutations showed an in-frame deletion in L71 gene, a collagen-like protein that eliminated almost all of its collagen motif sequences. The resulting viruses revealed a significant reduction in their infection titer as well as substantially reduced virus yield. Hair deficient-infected amoebae also burst less readily. We suggest that a possible role of the fibrils is in virus release from its host and for its efficient production.

### Comparative Study of Biological Activities of Some Heterocyclic Compounds Derived from Spiro-Isoxazoline, Triazole, Tetrazole, Pyrazole, and Tryptophan 

AlamiAnouarSidi Mohammed Ben Abdellah University, Morocco; anouar.alami@usmba.ac.maThe chemistry of the heterocycles constitutes one of the research themes that is well studied and developed in organic synthesis. Many heterocyclic derivatives are found to exhibit various biochemical, agro-chemical, and electrochemical activities. In continuation of our research interest in heterocyclic compounds and those precursors, we report in this conference the latest research conducted in our Laboratory of Organic Chemistry. The research orientations chosen are the development of some spiro heterocyclic compounds, study and prediction of their pharmacological activities, and synthesis and evaluation of the antibacterial activity of some heterocycles derived from triazole, tetrazole, pyrazole, and tryptophan.

### New Concentration Methodology for the Quantitative Detection of Viruses in a Drinking Water Large System

PagesGemma Saucedo^1^*JuanuixCarles Vilaró^1^CantosMaria Jose Arnedo^1^ColaciosEva Duran^1^PorcarBelen Galofré^1^AnfrunsEduard^2^FuentesCristina^2^BoschAlbert^2^LucenaFrancisco^2^1Aigues de Barcelona, Spain2University of Barcelona, Spain*Correspondence: gsaucedo@aiguesdebarcelona.catA new concentration methodology developed within the Aquavalens Project has been implemented in a routine control of a drinking water treatment plant (DWTP) and distribution network (DN). Samples were taken monthly during one year and a half at different treatment steps of the DWTP including raw water, sand filter, granulated active carbon, and treated water and also from distribution points representing different water origins. The employed methodology is based on large-volume concentration (from over 1000 L for distribution or treated water samples to 10 L for raw water) by using a commercial ultrafiltration filter (Rexeed© 25A). After adsorption, viruses were eluted from the filter in 500–600 mL that were further concentrated by using Centricon©, and eluted again in 2–4 mL. Genetic material is extracted and analyzed by q(RT)-PCR for hepatitis A virus, noroviruses GI and GII, and adenoviruses, and by an integrated cell culture-PCR for enteroviruses and rotaviruses. Results were obtained during 2018 and 2019. Positive samples were mainly found in raw water and first treatment steps, such as sand filters, and no positive results were detected in treated and distribution samples. These results were compared to those from previous years, when samples were concentrated by adsorption-elution using electropositive filters (Zeta Plus). The number of positive samples was quite similar for each treatment step but quantification was higher than in previous years. Higher recoveries for viruses have already been ascertained within the framework of the Aquavalens Project when this new method was evaluated. The employed methodology provides new tools for the monitoring of health-significant viruses in the water environment, enabling to perform quantitative microbial risk assessment analysis, altogether intended to be used in the development of risk management strategies. Such strategies, if properly implemented, will ensure the application of a Water Safety Plan in a duly diligent manner such that reasonably foreseeable harm is identified, prevented, and sound measures are taken to protect the consumer.

### Cyanobacterial Lipopolysaccharide Pro-Inflammatory Potency Can Be Underestimated by Standard Endotoxin Test

SindlerovaL.^1^*VasicekO.^1^BabicaP.^2^MoosovaZ.^1^,^2^HosekovaV.^1^,^2^GoliasovaZ.^1^,^2^1Academy of Sciences of the Czech Republic, Czech Republic2Masaryk University, Czech Republic*Correspondence: sindler@ibp.czHarmful algae blooms (HAB) are a source of lipopolysaccharides (LPS) which are released into the water during cell division and lysis. Bacterial LPS is known to be a potent pro-inflammatory agent but the cyanobacterial LPS is not studied well. To study its ability to induce pro-inflammatory responses, LPS extracts from HAB biomasses dominated by different species as well as from axenic and non-axenic cultures of the same species were prepared. First, pyrogenicity of these LPS was tested using Pyrogene assay. There was a wide range of pyrogenic activity among all samples, from dozens to millions of EU/mg LPS. Further, production of pro-inflammatory cytokines was detected using murine macrophages RAW264.7 and human intestinal epithelial cells Caco-2 and HT-29. In intestinal cells, level of interleukin 8 was significantly increased also after exposure to LPS from HAB with low pyrogenicity. Moreover, LPS from axenic culture of *Aphanizomenon flos-aquae* PCC7905 and *Planktothrix agardhii* PCC7805 significantly increased levels of tumor necrosis factor α in macrophages despite the fact that their pyrogenicity was very low. Since LPS is generally recognized as a ligand of TLR4, its antagonist was used but it did not affect pro-inflammatory effects of studied LPS. We can conclude that pro-inflammatory potency of cyanobacterial LPS may not be predicted by Pyrogene test and can be significantly underestimated. Moreover, we showed that not only environmental mixtures but also pure cyanobacterial LPS can exert significant biological properties possessing risk to human health. Further, these effects seem not to depend on TLR4.

## Video Presentation

### Effects of Arginine Supplementation on Antler Growth, Serum Biochemical Indices, and Rumen Bacteria Community of Sika Deer (Cervus nippon)

LiZhipeng^1^*SiHuazhe^1^NanWeixiao^1^LiuHanlu^1^JinChunai^1^LiGuangyu^1^LouYujie^2^1Chinese Academy of Agricultural Sciences, China2Jilin Agricultural University, China*Correspondence: zhplicaas@163.comVelvet antler is the only regenerating organ in mammals, which is an important Chinese medicine generated from Sika deer (*Cervus nippon*). It is estimated that approximately 800,000 heads of sika deer are farmed in China for production of velvet antlers. The metabolism of arginine is related to the production of velvet antlers. However, the underlying mechanism remains unclear. To investigate and understand the effects of arginine supplementation in the diet on antler production, serum metabolic parameters, rumen fermentation, and bacterial community of sika deer during growth period were examined. A total of 15 male sika deer (6-year-old) were randomly assigned to three dietary treatments, which were supplemented with 0 (n = 5, AR), 2.5 (n = 5, BR), or 5.0 g/d (n=5, CR) L-arginine, respectively. At the end of the experiment, velvet antlers were sawed and weighed, the concentration of serum parameters, and volatile fatty acids (VFAs) in rumen were measured. The rumen bacterial community based on the V3-V4 region of 16S rRNA gene was also examined. The BR group has the highest weight of velvet antlers among the three groups. The amounts of IGF-1, alanine aminotransferase, and aspartate aminotransferase in serum were significantly greater in the BR group than those in AR and CR groups. The concentrations of acetate, propionate, butyrate, isobutyrate, isovalerate, and valerate were also significantly higher in the BR group than those in the other three groups. Moreover, the bacterial diversity indices including the OTU numbers, ACE and Chao1 indices in BR and CR groups increased in comparison with that in the AR group. In addition, arginine supplementation caused the increase of *Fibrobacter* spp. and *Prevotellaceae_UCG-003*, and the decrease of *Corynebacterium_1* and *Clostridium_sensu_stricto_1*. Moreover, the BR group showed the significantly highest abundance of *Bacteroides* among the three groups. These results demonstrated that arginine supplementation affected the metabolism and microbiota in rumen of sika deer. 

## e-POSTERS

### Bio-Management of Citrus Nematode, Tylenchulus semipenetrans and Dry Root Rot Fungi, Fusarium solani under Laboratory and Field Conditions

IbrahimDina S. S.*AliAyat M.MetwalyHowida A.Agricultural Research Center, Egypt*Correspondence: mickeyocean@yahoo.comSlow decline and dry root rot of citrus trees caused by *Tylenchulus semipenetrans* and *Fusarium solani*, respectively, are serious diseases attacking many groves in Egypt. Efficacy of the bio-agents (*Trichoderma harzianum*, *Bacillus subtilis*, *Streptomyces griseus*, and *Paecilomyces lilacinus*) against citrus nematode *T. semipenetrans* and their combination with Nemastop (natural oils) were studied in vitro and in vivo. Such bio-agents and Nemastop were also studied under field conditions against both *T. semipenetrans* and *F. solani* infecting Washington Navel orange trees (*Citrus aurantium *L.) compared with control and Nemaphos. To determine the inhibition effect of Nemastop and different bio-agents, on *T. semipenetrans *in vitro and in vivo*,* we studied the effect of Nemastop (natural oils) and four bio-agents i.e., *B. subtilis*, *T. harzianum, S. griseus*, *P. lilacinus *on survival of *T. semipenetrans *juveniles under laboratory conditions. Research was carried out in a private orchard during 2017 and 2018 seasons on 10 years old Washington Navel orange trees, (*Citrus aurantium *L.) naturally infested with *T. semipenetrans*. In vitro data revealed that all tested bio-agents had various degrees of effectiveness towards the juvenile's survival compared with control treatment. Meanwhile, Bio-Nematon achieved the highest percentage of mortality for *T. semipenetrans* (50.0%). The population and incidence of soil borne pathogens were examined after 3, 7 and 12 months in vivo. Results showed that all treatments led to a clear significant reduction in disease incidence compared with control treatment. All tested bio-agents gave a good effect on decreasing the *T. semipenetrans* population and *F. solani* incidence for 12 months. A clear significant decrement in *T. semipenetrans* population was noticed with the combination of *T. harzianum* with Nemastop. Meanwhile, *B. subtilis* or *T. harzianum* reduced *F. solani* incidence. The effect of different bio-agents singly or integrated with Nemastop on *T. semipenetrans *survival was determined in vitro and in vivo. All tested bio-agents had various degrees of effectiveness toward *T. semipenetrans *compared with the control treatment.

### Augmentation of Dietary Glucosylceramide Utilization by Probiotic Bacteria

AsanumaNaritoMeiji University, Japan; asanuma@meiji.ac.jpThe major sphingolipids included in plant food are glucosylceramide (GluCer). The intermediates of GluCer metabolism, such as ceramides and sphingoid bases, are known to exert various biological functions. However, dietary GluCer is barely hydrolyzed to ceramide in the intestinal tract, since the intestinal enzyme activity of glucosylceramidase is extremely low. Thus, it is important to enhance GluCer hydrolysis by intestinal bacteria to improve the utilization of dietary GluCer. We isolated the novel bacteria that hydrolyzes GluCer and examined their probiotic effects for GluCer digestion. Bacteria that hydrolyze GluCer were sought from feces collected from various animals. Colonies of each culture were obtained by the Hungate roll-tube method. The bacteria from the colonies were cultured to test for GluCer-hydrolyzing ability. To examine probiotic effects of the isolated bacteria, live cells were administered to mice using a stomach tube at a level of 10^9^ cfu/mouse/day. Lipids were extracted from each sample of diets or feces with chloroform-methanol (2:1, v/v), and GluCer was separated by thin-layer chromatography. Two GluCer-hydrolyzing bacteria (Bacterium A and B) were isolated from the canine feces. When these isolated bacteria were grown in GluCer-containing medium, GluCer was hydrolyzed and ceramide was accumulated in each culture. The GluCer-hydrolyzing activity of Bacterium A was much higher than that of Bacterium B. When apple powder was fed to mice as GluCer-containing food material, some amount of GluCer was excreted in feces. However, administration of Bacterium A cells significantly decreased the amount of GluCer recovered in feces. The utilization of GluCer-hydrolyzing bacteria as probiotics may be useful to augment GluCer digestion in the large intestine.

### Antibiotic Resistance among Uropathogenic Escherichia coli Isolated from Children with Congenital Malformations of the Urinary System in Moscow Russia

S.Souadkia*I. V.PadaprigouraI.KhelifiPeople’s Friendship University of Russia, Russia*Correspondence: sarah1sdk@gmail.comThe length of telomeric DNA is maintained by the enzyme telomerase. However, to control this process, a number of accessory proteins are involved, including six telomere-associated proteins: hTRF1 (human telomeric repeat-binding factor 1), hTRF2 (human telomeric repeat-binding factor 2), POT1, RAP1, TIN2, and TPP1. It was shown that these proteins in mammalian cells form a DNA-protein cluster, known as a shelterin complex. The complex is essential for a telomere function. It ensures the stability of the chromosome by remodeling the telomeric DNA into a specific t-loop structure and protects the telomeric DNA against undesirable activity of the DNA repair pathway. Both hTRF1 and hTRF2 exhibit high affinity for a double-stranded telomeric DNA and bind to DNA as homodimers. The TIN2 is a key component of the complex and associates with both TRF1 and TRF2. TRF1-TIN2 interaction occurs through the TRFH domain and the TIN2 carboxy-terminal domain. TIN2 is essential for bringing together the DNA-binding proteins within the shelterin complex. Evaluation of the recombinant TRF1/2 and TIN2 protein usefulness in study of the protein-peptide, protein-DNA interaction in vitro. The developed, simplified model of the shelterin complex will be used for testing of several chemical compounds, which may be potential anti-cancer drug candidates. We designed codon-optimized and expressed synthetic genes encoding the selected proteins from the shelterin complex. Recombinant proteins were purified by affinity chromatography methods. Protein-protein and protein-DNA interactions were studied by SDS-PAGE and EMSA analysis. Synthetic genes, coding for recombinant, full-length hTRF1/2 proteins as well as their isolated DNA binding domains (Myb1/Myb2) were overexpressed in the *Escherichia coli* system and the proteins were purified. In addition, short peptide fragment of TIN2 was synthesized. Its role in forming of the specific DNA-TRF1/2 complex was evaluated. The ability of the obtained, recombinant protein variants to bind selectively to a telomeric DNA was confirmed.

## Poster Presentation

### Study of the Interaction between Recombinant, Shelterin Proteins and Telomeric DNA

Zylicz-StachulaAgnieszka*ŻebrowskaJoannaPrusinowskiMaciejLaszukPatrycjaKrefftDariaSobolewskiIrekMaciejewskaNataliaLubkowskaBeataStruckAnnaSkowronPiotr M.University of Gdansk, Poland*Correspondence: a.zylicz-stachula@ug.edu.plThe length of telomeric DNA is maintained by the enzyme telomerase. However, to control this process, a number of accessory proteins are involved, including six telomere-associated proteins: hTRF1 (human telomeric repeat-binding factor 1), hTRF2 (human telomeric repeat-binding factor 2), POT1, RAP1, TIN2, and TPP1. It was shown that these proteins in mammalian cells form a DNA-protein cluster, known as a shelterin complex. The complex is essential for a telomere function. It ensures the stability of the chromosome by remodeling the telomeric DNA into a specific t-loop structure and protects the telomeric DNA against undesirable activity of the DNA repair pathway. Both hTRF1 and hTRF2 exhibit high affinity for a double-stranded telomeric DNA and bind to DNA as homodimers. The TIN2 is a key component of the complex and associates with both TRF1 and TRF2. TRF1-TIN2 interaction occurs through the TRFH domain and the TIN2 carboxy-terminal domain. TIN2 is essential for bringing together the DNA-binding proteins within the shelterin complex. Evaluation of the recombinant TRF1/2 and TIN2 protein usefulness in study of the protein-peptide, protein-DNA interaction in vitro. The developed, simplified model of the shelterin complex will be used for testing of several chemical compounds, which may be potential anti-cancer drug candidates. We designed codon-optimized and expressed synthetic genes encoding the selected proteins from the shelterin complex. Recombinant proteins were purified by affinity chromatography methods. Protein-protein and protein-DNA interactions were studied by SDS-PAGE and EMSA analysis. Synthetic genes, coding for recombinant, full-length hTRF1/2 proteins as well as their isolated DNA binding domains (Myb1/Myb2) were overexpressed in the *Escherichia coli* system and the proteins were purified. In addition, short peptide fragment of TIN2 was synthesized. Its role in forming of the specific DNA-TRF1/2 complex was evaluated. The ability of the obtained, recombinant protein variants to bind selectively to a telomeric DNA was confirmed.

### Lack of Vitamin B12 Synthesis in Mycobacterium tuberculosis Grown under Various Conditions

MiniasAlina^1^*CzubatBożena^1^,^2^DziadekJarosław^1^1Institute of Medical Biology, Polish Academy of Sciences, Poland2University of Rzeszów, Poland*Correspondence: aminias@cbm.pan.plVitamin B12 (cobalamin) is a water-soluble vitamin that affects mycobacterial metabolism through acting as a cofactor of the enzymes or through binding to riboswitches that control gene expression. The reference strain of* M. tuberculosis* H37Rv was reported not to synthesize cobalamin during culture-rich broth in various indirect experiments. Such a result is surprising given that the genome of the bacterium contains genes necessary for the biosynthesis pathway. We wanted to test whether clinical strains of *M. tuberculosis* might synthesize vitamin B12 or whether vitamin could be synthesized under specific environmental conditions using de novo or salvage pathways. We used immunoenzymatic assay to determine vitamin B12 concentration per mg of protein in whole-cell lysates of cultures of laboratory strain H37Rv and five clinical strains, each belonging to distinct spoligotype lineages. As controls, we used strain ∆*cobIJ *deficient in *cobIJ* gene involved in vitamin B12 biosynthesis pathway and *Mycobacterium smegmatis* mc^2^. The cultures were grown in rich medium, poor medium, under acidic conditions, and under hypoxic conditions. Further, we tested persister cultures and cultures supplemented with uroporfirynogen III. We did not detect vitamin B12 in any cell lysates obtained from *M. tuberculosis* cultures, in contrast to *M. smegmatis*, which logarithmic phase cultures on average contained 3.8 ± 0.38 ng of vitamin B12 per mg of protein. We conclude that *M. tuberculosis* is not able to synthesize vitamin B12 de novo under conditions tested.

### Population Structure and Patterns of Genetic Variation in a Pearl Oyster Pinctada radiata Native to the Arabian Gulf

Al-SaadiAmal MohammedMinistry of Municipality and Environment Doha, Qatar; alsaadi@email.comThe pearl oyster *Pinctada radiata*, a marine species native to the Arabian Gulf in the Indian Ocean is common in Qatari waters where it comprises 95% of pearl oyster stocks found naturally there. This species has been harvested for many centuries primarily for natural pearls but also for edible flesh and a lustrous shell*. P. radiata* possesses a relatively long-lived pelagic larval phase and adults at least, can tolerate hypersaline conditions. As sedentary relatively long-lived bivalves, pearl oyster species have the capacity in general, to accumulate metallic pollutants in their soft tissues to levels higher than surrounding background levels in seawater*. P. radiata* therefore, has been used extensively as a natural bio-indicator for monitoring pollution in the Arabian Gulf. Sustainability of *P. radiata* wild stocks are now significantly threatened in the Arabian Gulf region as a result of both natural (e.g., extreme salinity, high temperatures, high evaporation rates, and low flushing rates) and anthropogenic factors (e.g., rapid development of coastline areas, overfishing, and heavy exploitation). Long-term sustainability and conservation of this local resource in the Arabian Gulf has therefore become a serious issue, but their survival can significantly contribute to maintenance of healthy marine ecosystems across the region. The current study assessed natural levels and patterns of genetic variation in Arabian Gulf populations of the native pearl oyster, *P. radiata* to define wild population structure. Potential intrinsic (e.g., pelagic larval phase and life history traits) and extrinsic factors (e.g., water current, wave action, water temperature, and prevailing wind direction) that could influence wild population structure were investigated. MtDNA (COI) sequences were used here to define levels and patterns of genetic diversity within and among three sample sites in Qatar territorial waters. Results of statistical analysis that partitioned variation (AMOVA) showed that virtually all variation was present within *P. radiata* sampled sites (94.61%) and differentiation among sites was relatively low (5.39%). Shallow differentiation of *P. radiata* wild populations in Qatar can largely be attributed to high levels of ongoing gene flow that results from dispersal mediated by a relatively long pelagic larval phase. Wild populations of *P. radiata* sampled from the Qatari coast were panmictic and therefore should be considered to constitute a single management unit. The present study was also the first to generate a massive GSS dataset using NGST for the Arabian pearl oyster *P. radiata*, the objective being primarily to identify microsatellite markers (SSRs) with potential to be applied in future population genetic studies after the failure of PCR cross-amplification trials with primers sets developed for other *Pinctada* species. The GSS dataset generated a large number of putative microsatellite motifs for *P. radiata* that may be useful in future population genetic studies. In addition, a number of putative genes were also identified after bioinformatics analysis with potential functional roles in toxicological and/or environmental stress responses. Some putative genes identified could also influence growth and survival of the target species. The partial genome (GSS) dataset for *P. radiata* potentially can have future applications in both pearl oyster farming and biotechnology.

### The Mycobacterial RadA and DisA Proteins Could Participate in Repair of DNA Damages

BrzostekAnna^1^*PłocińskiPrzemysław^1^PłocińskaRenata^1^Korycka-MachałaMałgorzata^1^CiszewskaAneta^1^GąsiorFilip^1^,^2^DziadekJarosław^1^1Institute of Medical Biology, Polish Academy of Sciences, Poland2University of Lodz, Poland*Correspondence: a.brzostek@cbm.pan.pl*M. tuberculosis* (*Mtb*) belongs to the most dangerous bacterial pathogens, responsible for 1.5 million deaths each year. Several proteins implicated in DNA damage repair, as well as proteins involved in lipid biosynthesis, proteases, transporters, cell wall organisation and synthesis, and signal transduction in mycobacteria, are known to influence bacterial cell survival during infection in animal models of tuberculosis, and are thus regarded as virulence factors. *Mtb* is exposed to oxygen and nitrogen radicals, generated by the immune response cells causing its DNA damage. Major DNA damaging events require a well-coordinated response to promote cell's survival like SOS response, with RecA as key recombinase. To characterize the role of RecA paralogues-RadA and, its interaction partner, DisA and to clarify their involvement in generating RecA independent response to DNA damage, the global RNA sequencing and whole cell proteomics where used to identify RecA independent response of *Mtb*. The mutants lacking functional *disA* and *radA* genes were constructed using homologous recombination technique. The proteins (DisA, RadA, and RecA) were purified by affinity chromatography. It was found RadA and co-expressed DNA damage scanning protein-DisA, are between the key elements of RecA independent response. The growth analysis of mutants defective in synthesis of DisA and RadA, in the presence of mutagens (MMC, H_2_O_2_, MMS, UV), were performed. The interactions between RadA, DisA, and RecA proteins were studied with different methods (BTH, pull down assay, microscale thermophoresis). The RNA seq analysis identified DisA and RadA proteins in RecA independent response. Although, no significant differences were observed in survival of mutants in the presence of mutagens compared to control *Mtb* strain. In vitro interaction between RadA, RecA, and DisA proteins was proved. 

### Investigation of Natural Saccharomyces Hybrids from a Hungarian Vineyard

GerőcsAnnamária*Nemes-BarnásKatalinMájerJánosSzőkeBarnaMagalhãesFredericoGibsonBrianOlaszFerencNational Agricultural Research and Innovation Centre, Hungary*Correspondence: gerocs.annamaria@abc.naik.huMembers of the *Saccharomyces *genus can form interspecific hybrids, potentially combining desirable features of the parental strains, which is useful in wine making. To compare the properties of hybrids to representative parental strains. We isolated previously 23 strains from fermented grape juice and these isolates were identified with species-specific PCR and separated based on their interdelta PCR pattern. The newly identified hybrids, two parental type strains (*S. paradoxus* C850, *S. kudriavzevii* C950), a wild *S. cerevisiae* parental strain, and a commercially available starter (*S. cerevisiae* × *S. bayanus*) were investigated in laboratory-scale fermentations with synthetic must (200 g/L glucose) at 20 °C. Vinification properties of strains were monitored and after two weeks, ethanol concentration, pH, specific gravity, and residual sugar content of the final products were analyzed. We tested ethanol tolerance (at 10%, 12% v/v) and growth ability at different temperatures (5, 10, 16, 20, 37 °C). Our results showed that all isolates belonged to the *Saccharomyces* genus and that two strains were interspecific hybrids (*S. cerevisiae* ×* S. kudriavzevii* and *S. cerevisiae *×* S. paradoxus*). The 23 isolates showed 11 different interdelta patterns and all tested yeasts could grow in the presence of 12% ethanol and at 20 °C. The *S. cerevisiae* strain and the two hybrid strains could tolerate 16 °C better than the two other parental strains. The *S. cerevisiae* ×* S. kudriavzevii *hybrid showed faster fermentation than the parental yeasts. The fermentation effect of the* S. cerevisiae *×* S. paradoxus *hybrid was similar to the starter, however, the most effective strain was *S. paradoxus*. Every strain could ferment glucose completely. Analytic parameters of all wines were very similar, i.e., final ethanol concentration was between 9.9% and 10.7%. All hybrid yeasts are suitable for wine making and fermentation ability of hybrids is similar to the starter strain investigated. Further investigations will determine the different sensory profiles of wines generated with the different strains.

### Evolution and Spread of Tetracycline Resistance in Mycoplasma hominis Tunisian Strains

AbdelmoumenBoutheina Ben*BoujemaaSafaKadhraouiNadineMlikMardassi BéhijaUniversité de Tunis El Manar, Tunisia*Correspondence: boutheina.mardassi@pasteur.tn*Mycoplasma hominis* has an etiological role in genitourinary tract infections and appears to be associated with human infertility. In recent years, the resistance of *M. hominis* to antibiotics has been shown to have an increasing trend. The prevalence of this pathogen and the extent of its antibiotic resistance profiles vary geographically and may be related to local antibiotic use regulation. Thus, antimicrobial surveillance should be diligent and thorough for effective antimicrobial therapy, and to monitor the spread of resistant strains. To our knowledge, data about the phylodistribution of drug resistance in *Mycoplasma hominis* are very scarce. The aims of this study were to assess the antimicrobial susceptibility of 65 *Mycoplasma hominis* clinical strains recovered from Tunisian patients over 18 years, to identify the molecular basis of antibiotic resistance, and to investigate the phylogenetic relationships of resistant strains. Sixty-five clinical isolates were characterized using an Expanded Multilocus Sequence Typing (eMLST) scheme, including 10 genes (*uvrA, gyrB, ftsY, tuf, gap, p120’, vaa, lmp1, lmp3, p60*). Antimicrobial susceptibility of nine antimicrobial agents (tetracycline, doxycycline, ofloxacin, ciprofloxacin, levofloxacin, moxifloxacin, erythromycin, azithromycin, and josamycin) was determined using a broth microdilution method. Fluoroquinolones, doxycycline, and josamycine were found to be the most effective antibacterial agents. However, 22 strains belonging to 11 expanded multilocus sequence types (eSTs) proved resistant to tetracycline. The majority of these eSTs were genetically related, indicative of clonal expansion of tetracycline resistance. The present study provides relevant information on the antibiotic susceptibility of Tunisian *M. hominis* clinical strains, lending support to a clonal transmission of tetracycline resistance. This is likely to have an important implication in monitoring the spread of drug resistance among *M. hominis*. 

### The Contribution of MSMEG4305 Protein in Vitamin B_12_ Biosynthesis in Mycobacterium smegmatis

CzubatBożena^1^,^2^*MiniasAlina^1^DziadekJarosław^1^1Institute of Medical Biology, Polish Academy of Sciences, Poland2University of Rzeszow, Poland*Correspondence: b.czubat@gmail.com*msmeg4305 M. smegmatis* is a gene coding a two-domain protein. Sequence similarity analysis indicates that the C-terminal domain is the CobC domain, predicted to be involved in the synthesis of vitamin B_12_, while the N-terminal domain encodes an RNase H. There are two ways of utilization of propionate in *M. smegmatis:* through the methyl citrate pathway or through the methylmalonyl pathway which is dependent on the presence of vitamin B_12 _in cells. The aim of this project is to determine the possible role of MSMEG4305 protein in vitamin B_12_ synthesis in *M. smegmatis* cells. The ability to synthesize the B_12_ molecule by *M. smegmatis* was determined through the analysis of propionate metabolism. We compared the growth of wild type and mutants, *∆msmeg4305* and *∆msmeg3873* (as a negative control), on the minimal medium supplemented with propionate. All strains have additional deletion in *prpR* (*msmeg6643*) which regulates genes involved in the methyl citrate pathway. As a control we also analyzed the growth of all deficient mutants on minimal medium with glucose. We observed inhibition of growth *∆msmeg3873/*Δ*msmeg6643* mutant of *M. smegmatis* during all experiments. The strain of *∆msmeg4305/*Δ*msmeg6643 M. smegmatis *showed a significant decrease in growth from 24 to 48 h of culture on medium supplemented with propionate. Our results suggest that MSMEG4305 does influence the level of vitamin B_12_ synthesis in *M. smegmatis*, though further research is needed to confirm our hypothesis.

### Thermophilic Bacteriophage TP-84 Endolysin Identification, Isolation, Characterization, and Molecular Gene Cloning as well as Its Potential Application in Biotechnology

ZebrowskaJoanna*KrawczunNataliaCzajkowskaEdytaKrefftDariaSobolewskiIreneuszŁubkowskaBeataZylicz-StachulaAgnieszkaSkowronPiotrUniversity of Gdansk, Poland*Correspondence: asia.zebrowska87@gmail.comThermophilic bacteriophages are rarely studied and none of their life cycles have been deciphered to a degree similar to *Escherichia coli *viruses such as λ, T4, T7, or M13. Nevertheless, they are interesting objects for studying determinants of thermophilicity and for practical aspects of biotechnology processes using high temperatures. The project involved experimental and bioinformatics research concerning TP-84 bacteriophage genome and coded proteins. Its 47.7-kbp double-stranded DNA genome revealed the presence of 81 coding sequences (CDSs), coding for polypeptides of 4 kDa or larger. Interestingly, all CDSs are oriented in the same direction, pointing to a dominant transcription direction of one DNA strand. Based on a homology search, a hypothetical biological function could be assigned to 31 CDSs. One of those was the endolysin TP84_28 protein. It contains two domains: N-terminal domain responsible for the enzymatic activity and C-terminal domain that allows for specific binding to the selected sugars of the bacterial cell wall. The native, thermophilic bacteriophage TP-84 endolysin was purified to functional homogeneity and characterized. The endolysin encoding gene TP84_28 was cloned into *Escherichia coli* and a preliminary gene expression analysis was conducted. This confirmed the endolysin biological (lytic) activity against thermophilic host bacterium *G. stearothermophilus* host (Gram-positive) as well as mesophilic pathogenic Gram-negative bacteria. Endolysins are involved in lysis of the bacterial cell wall from the inside, releasing mature bacteriophages. Purified endolysins can be also used to lyse bacteria from the outside. Potentially, these enzymes can be used as a replacement for antibiotic therapy or for bacterial lysis for biotechnology purposes.

### Fabrication of Symbiotic Multicellular Assemblies by Using a Novel “Gel Layer-by-Gel Layer” Technique

Al-HaidoseReemMinistry of Municipal and Environment Doha, Qatar; ralhaidose@mme.gov.qaThe research on biofilms has skyrocketed in recent years due to increased awareness of the pervasiveness and impact of biofilms on natural and industrial systems, as well as human health. A biofilm is a well-organized, cooperating community of microorganisms. Microbial cells attach to the surfaces and develop a biofilm. The yeast species *Saccharomyces cerevisiae* is capable of forming biofilms on a variety of inert and biological surfaces. Cells in biofilms display phenotypic properties that are radically different from their free-floating planktonic counterparts, including their recalcitrance to antimicrobial agents. In this study, we described a simple, fast, inexpensive and highly reproducible formation of substrate based yeast biofilms by employing a novel “gel layer-by-gel layer” method based on the gelling properties of alginate gels. We combined two different types of cells, i.e., yeast and Chlorella (algae) cells to produced symbiotic two-layered biofilms by using a similar technique. We also include some preliminary results on free standing biofilms in solution which were produced by cleaving of patterned biofilms from the substrate. We demonstrate that the cells preserve their viability upon preparation and manipulation of these artificial biofilms. 

### Investigation of Lactic Acid Bacteria Isolated from the Gastrointestinal Tract of Wild Boars

KeresztényTibor*LibischBalázsVitányiBeátaKerényiZoltánKocsisRóbertOlaszFerencPappPéterNational Agricultural Research and Innovation Centre, Hungary*Correspondence: kereszteny.tibor@abc.naik.huDuring the last decade it has become widely accepted that balanced microbiota with substantial diversity is essential for healthy life of living organisms within the animal kingdom. Disruption of this equilibrium is linked with the development of different diseases. However, it has been also recognized that beneficial microorganisms can help to prevent or alleviate the symptoms of microbiota disruptions and even can promote growth and yield of livestock production. The aim of this study was to characterize lactic acid bacteria (LAB) isolated from wild boars *(Sus scrofa)* regarding their in vitro antimicrobial activity, bile salt and acid tolerance, in order to find potential candidates which can be efficiently used as feed additives in the swine industry. Samples were collected from four different sections of the gastrointestinal tract (ileum, cecum, colon, and rectum) of free-living wild boars. Microorganisms were isolated from the samples using specific culture media, then the isolates were screened by using genus-specific PCR primers and some of them were identified at species level by sequencing of their 16S rRNA gene. Antimicrobial activity of the selected LAB isolates was assayed by agar-well diffusion method. Bile salt and acid tolerance characterization are in progress by using specific culture media for evaluation, supplemented with bile salt and HCl, respectively. The majority of the examined isolates had antibacterial activity against at least one of the used indicator strains (*Escherichia coli *K12*, Staphylococcus aureus *SU17, *Salmonella enterica *serov. Typhimurium LT2, and *Streptococcus thermophylus *T9). Isolated *Lactobacillus mucosae, Leuconostoc mesenteroides*, and* Streptococcus hyointestinalis* strains with high bile salt and acid tolerance may have beneficial features. Our results demonstrate the ability of some LAB strains isolated from the gastrointestinal tract of wild boars to inhibit the growth of potential pathogenic bacteria. These isolates are scheduled for further investigations, including animal feeding studies, to develop suitable feed additives to prevent in vivo gastrointestinal infections and to promote higher yields in the swine industry.

### Comparison of Eight Methods for Metagenomic DNA Extraction Suitable for Next Generation Sequencing (NGS) Technologies Using in Vitro Colonic Fermentation Samples

Ruíz-ValdiviezoVíctor Manuel^1^*Peña-OcañaBetsy Anaid^1^Guriérrez-SarmientoWilbert^1^Sáyago-AyerdiSonia Guadalupe^2^1Tuxtla Gutierrez Institute of Technology, Mexico2Tepic Institute of Technology. Mexico*Correspondence: bioqvic@hotmail.comIn vitro colonic fermentation is a widely used methodology for understanding the changes of intestinal microbiota by food nutrients. Although there are diverse methods for DNA isolation of different samples, there is not a standard method employed for this kind of experimental sample in a complex mix that included substrates food, fecal material, enzymes, and simulated intestinal fluid. To evaluate the efficiency and quality of eight different metagenomic DNA extraction methods from in vitro colonic fermentation samples Methods 1, 2, and 3 were direct applications of three commercially available kits, ZymoBIOMICS™ DNA Miniprep Kit, ZR Fecal™ DNA MiniPrep Kit, DNEasy™ Blood & Tissue Hanbook, and standardized methods (4) EDTA-Tris-HCl (Valenzuela-Encinas et al., 2008), (5) Triton-SDS (Winstoff and Hoffman, 1987), (6) Lysozyme (Sambrook and Russell, 2001), (7) Urea (Yalçınkaya et al., 2017), and (8) Sal method (Yalçınkaya et al., 2017). The integrity of DNA isolated was confirmed in agarose gel electrophoresis, while the quantity and quality were measured in Nanodrop One (Thermoscientific, USA). The agarose gel images revealed the presence of metagenomic DNA for all extraction methods. Results revealed differential significant in DNA among the eight methods. ZymoBIOMICS™ 66.67 ± 21.74 ng/µL, ZR Fecal™ 29.59 ± 0.31 ng/µL, DNEasy™ Blood & Tissue Hanbook 0.72 ± 9.69 ng/µL, EDTA-Tris-HCl 35.75 ± 5.04 ng/µL, Triton-SDS 33.97 ± 7.26 ng/µL, Lysozyme 67.18 ± 5.13 ng/µL, Urea 40.40 ± 1.25 ng/µL, and Salt 32.18 ± 2.03 ng/µL, while the 260/280 ratios were 1.71, 1.84, 1.47, 2.38, 2.49, 2.33, 2.04, 2.34, respectively, as a quality DNA. The quantity and quality of DNA obtained showed significant variation among the eight methods. For this type of sample, the most efficient methods were ZymoBIOMICS™ commercial kit (Method 1) and standardized method 6, which can be archived for future application of next generation sequencing (NGS) technologies.

### Efficient Enrichment of Prokaryotic RNA from Active Volcano Samples: Lab-Lake Culture System

Peña-OcañaBetsy Anaid*^1^Ruíz-ValdiviezoVíctor Manuel*^1^Gutiérrez-SarmientoWilbert^1^Jasso-ChávezRicardo^2^1Tuxtla Gutierrez Institute of Technology, Mexico2National Institute of Cardiology Ignacio Chavez, Mexico*Correspondence: poba15@hotmail.com; bioqvic@hotmail.comThe active volcano El Chichón in Chiapas (Mexico), is a dynamic poly-extreme site due to high temperature (30 and 95 °C), acidophilic pH (1-5), high salinity, high content of metals (Fe, Al, As, Cd), as well as low content of organic carbon. Recently, the presence of bacteria in the crater-lake was reported; however, the genetic and metabolic processes by which these microorganisms survive in this environment are still unknown. To evaluate the efficiency of the enriched cultures in a “lab-lake system” to increase the yield and quality of RNA extraction for meta-transcriptomic analysis of microbiome from the volcano El Chichón, the acid-thermic water was collected from the crater-lake and the geochemical characteristics were determined. An anaerobic culture system was employed and supplemented with glucose, maltose, sodium pyruvate, triacylglycerol, and glycerol as a carbon source for microbial enrichment in similar extreme conditions of the volcano. Total protein content and methane production were determined as evidence of cell growth. The total environmental RNA (eRNA) and enrichment system RNA (sRNA) content was extracted and quantified. A total of 15 lake samples were analyzed, these samples showed low C (1.7 ppm), high Na^+^ (3125 ppm), Cl^-^ (4484 ppm), Al (4875 ppm), Fe (3193 ppm), and As (140 ppm). The anaerobic growth system allowed a yield of 7 mg protein/60 mL cultures. The concentration of eRNA was 1.6 ng while sRNA increase to 6µg/60 mL cultures. The number of microorganisms present in the acid-thermic water of the crater-lake is low, among other factors, due to the low availability of nutrients; however, when anaerobic enrichment is supplemented with a mixture of sources of C, the growth of prokaryotic communities is favored. The anaerobic lab-lake microbial enrichment system is an efficient strategy to obtain an appropriate sRNA concentration and quality for future metatranscriptomic studies.

### Comparative Analysis of Shewanella xiamenensis LC6 Transcriptome during Degradation of Two azo Dyes

CarloMormontoyWendyLizarragaHederssonCallaMarioTairaPabloRamirez*National University of San Marcos, Peru*Correspondence: pramirezr@unmsm.edu.peTextile industry generates the largest volumetric amount of residual effluents. One of the most concentrated pollutants in textile effluents is the synthetic dyes used during dyeing. Recently, an efficient and suitable method for removing dyes has performed through bacterial biodegradation. Due to its great biodecolorization activity, the genus Shewanella has been studied intensively. To profile the *Shewanella xiamensis* LC6 transcriptome during decolorization of two azo dyes with different polarity, a comparative study of the *S. xiamenensis* LC6 transcriptome was performed while growing in minimal medium before (Control) and after challenged with two separate dyes: Methyl Red (MR) and Methyl Orange (MO) under microaerophilic conditions. cDNA (previously rRNA depleted) was sequenced by NovaSeq (Illumina^®^), insert length was 250 bp. From at least 4200 mapped genes, 417 differentially expressed genes (DEGs) were obtained under MO/Control conditions and 183 DEGs under MR/Control (log2FC> 1, FDR <0.05). A total of 112 genes were upregulated under both comparative conditions. Notably azoR, an azoreductase related to the specific cleavage of azo bond, was more intensely expressed under MR respect to MO exposure, suggesting its main intracellular activity. Additionally, genes coding for efflux pumps were overexpressed (bepE, bepF) suggesting greater intracellular stress during MR compared to MO conditions. On the other hand, during degradation of MO, the intensified use of Fe (as heme groups) would correspond to an intense activity of the decahaem cytochromes from the Mtr pathway (genes such as hutX, hutZ, and HxuC). Other pathways such as glucose metabolism (pgcA), tricarboxycyclic acid cycle (acnD), and urea cycle (argF) were altered. Finally, at least five hypothetical genes and one new transcript with unknown functions were overexpressed in both conditions. *Shewanella xiamenensis* LC6 uses both pathways during the decolorization process: azoreductase-mediated and extracellular electron transfer process. Although, the intensity of these responses will depend on the polarity of the dye.

### Physiological and Genomic Approaches of Shewanella algae and Shewanella xiamenensis in Decolorization of azo and Antraquinones Dyes

WendyLizárragaCarloMormontoyHederssonCallaMaríaCastañedaPabloRamirez*National University of San Marcos, Peru*Correspondence: pramirezr@unmsm.edu.peTextile industry frequently use azo dyes due to its high performance and low cost, despite being the most difficult group to degrade; described as a possibly mutagenic, carcinogenic, and recalcitrant agent. Given this problem, the genus Shewanella emerges as a possible bioremediating agent due to its versatility to use a wide variety of substrates as final electron acceptor. The present study aimed to identify genes involved in azo and anthraquinones dye degradation processes. The strains used were *Shewanella algae* 2NE11 and *Shewanella xiamenensis* LC6, to which growth kinetics and decoloration kinetics were performed. Dyes evaluated were Direct Blue 71, Methyl Orange, HEXL Proction Yellow, and Remazol Bright Blue at 100 mg/L. The total DNA of the two strains was sequenced by SMRT RSII and the genomes were assembled with Unicycler and annotated with Prokka. Genes related to decolorization were identified and aligned with ESPript. The phylogeny of azoreductases was obtained from Neighbor joining, Maximum likelihood, and Bayesian inference consensus with 10,000 bootstraps. Both strains reach 90% decolorization rate after 12 h of exposure to all dyes. Genome analysis allowed identification of the presence of an FMN-dependent NADH-azoreductase, FMN-dependent NADPH-reductase, and heme-dependent Dyp peroxidase in both strains suggesting that it would be related to decolorization process of azo and anthraquinones dyes as previously described. Analysis of alignment and phylogeny of aminoacidic sequence reveals that an ACP phosphodiesterase gene in *S. algae* 2NE11 is close related to azoR (<50% similarity). Additionally, the active site and c-terminal sequence are conserved, suggesting that it could encode a new type of azoreductases. *S. xiamensis* LC6 and *S. algae* 2NE11 decolorizate azo and anthraquinones dyes efficiently. An ACP phosphodiesterase gene in *S. algae* 2NE11 could belong to a new type of azoreductase not described previously. 

